# Unassigned diversity of planktonic foraminifera from environmental sequencing revealed as known but neglected species

**DOI:** 10.1371/journal.pone.0213936

**Published:** 2019-03-21

**Authors:** Raphaël Morard, Nele M. Vollmar, Mattia Greco, Michal Kucera

**Affiliations:** MARUM—Center for Marine Environmental Sciences, University of Bremen, Bremen, Germany; Universita degli Studi di Urbino Carlo Bo, ITALY

## Abstract

Most research on extant planktonic foraminifera has been directed towards larger species (>0.150 mm) which can be easily manipulated, counted and yield enough calcite for geochemical analyses. This has drawn attention towards the macroperforate clade and created an impression of their numerical and ecological dominance. Drawing such conclusions from the study of such “giants” is a dangerous path. There were times in the evolutionary history of planktonic foraminifera when all species were smaller than 0.1 mm and indeed numerous small taxa, mainly from the microperforate clade, have been formally described from the modern plankton. The significance of these small, obscure and neglected species is poorly characterized and their relationship to the newly discovered hyperabundant but uncharacterized lineages of planktonic foraminifera in metabarcoding datasets is unknown. To determine, who is hiding in the metabarcoding datasets, we carried out an extensive sequencing of 18S rDNA targeted at small and obscure species. The sequences of the newly characterized small and obscure taxa match many of the previously uncharacterized lineages found in metabarcoding data. This indicates that most of the modern diversity in planktonic foraminifera has been taxonomically captured, but the role of the small and neglected taxa has been severely underestimated.

## Introduction

Planktonic foraminifera are characterized by a modest diversity of ~50 morphologically defined species traditionally assigned to four clades [1,2]. Single-cell barcoding efforts of the past decades provided reliable barcodes for a substantial portion of the morphologically manifested diversity within each of these clades [3], paving the way for a new generation of metabarcoding surveys of the distribution of taxa in this group [4]. Since the diversity of planktonic foraminifera is based on diagnostic characters of their calcite shells, and the barcoding effort covered all of the abundant lineages, it was assumed that barcode sequences from environmental samples would be easily assigned to the existing taxonomic backbone. Therefore, it came as a surprise when the first metabarcoding survey [4] revealed abundant lineages of foraminifera occurring in small size fractions of the plankton that did not fit into the existing taxonomic framework. This discovery raised uncomfortable questions, with serious implications on our view of planktonic foraminifera diversity and community composition. Is it possible that taxonomers working on the plankton for over a century completely missed apparently abundant taxa? Could there be planktonic foraminifera that escaped detection because they lost their shell? Or, could it be that we have underestimated the role and diversity among the taxa for which we have not yet generated barcodes? Perhaps, the taxa that are not yet genetically characterized are not just variants of the existing diversity, but represent, unexpectedly, distinct lineages?

Since we consider it unlikely that abundant lineages of foraminifera in the plankton would have escaped detection, we decided to elucidate the nature of the uncharacterized lineages by extending the coverage of the barcodes. Specifically, we have followed a barcoding effort targeted towards small and obscure taxa of planktonic foraminifera and extended the coverage for the microperforate clade, which remains largely uncharacterized in terms of its cryptic diversity. We implemented the new DNA sequences into an objective molecular nomenclature framework [5] and used this to structure the barcoding reference into meaningful taxonomic units. We then used this reference to re-assign environmental barcodes of the analysis by Morard et al. [4] where the unassigned lineages were discovered, and extended the re-assignment to the global sampling of the Tara Oceans expedition V9 metabarcoding survey of de Vargas et al. [6], which has a global coverage.

## Material and methods

### Selection of material

**Fig 1:**
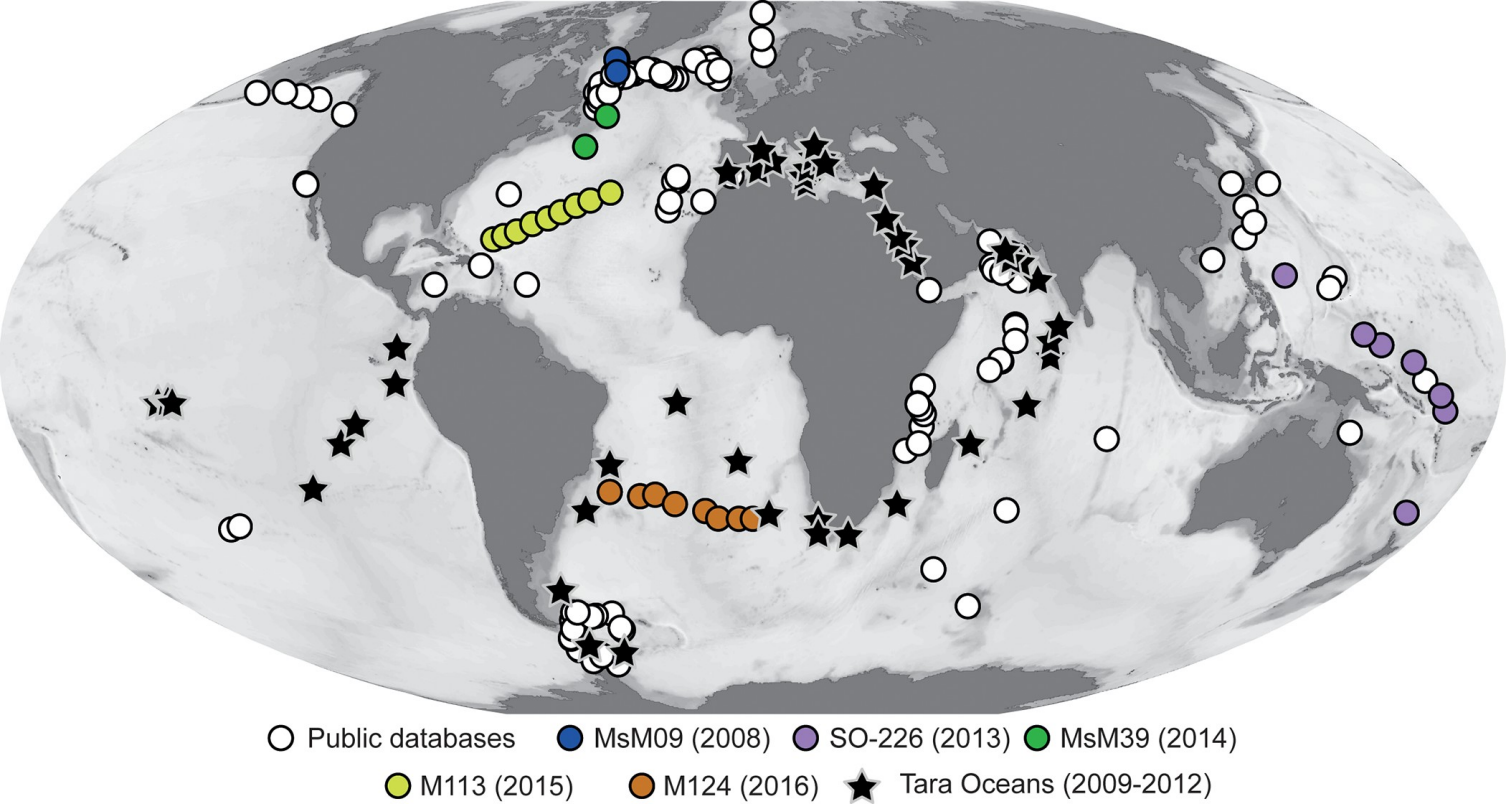
Location of the samples analyzed in the study. The circles indicate the single-cell collection and the stars the TARA Oceans stations. The map was generated using Ocean Data View [30].

To obtain barcodes for the previously genetically uncharacterized species of planktonic foraminifera, we took advantage of the large number of cryopreserved specimens available in our collections at the University of Bremen. Our collection contains specimens recovered from multi-net and ship-pump samples collected between 0 and 700 meters using nets with mesh sizes from 63 to 200 μm. The specimens isolated from the plankton were cleaned with brushes and either transferred onto cardboard slides and air-dried or isolated into either DOC or GITC* DNA extraction buffer (freshly prepared following procedures described in Weiner et al. [7]) and stored at -20°C or -80°C until further processing. Further details of the collection procedures are described in Weiner et al. [7] and the handling and extraction procedure used on each specimen is listed in S1 Table. To obtain new barcode sequences, we screened micropaleontological slides with dried planktonic foraminifera quantitatively isolated from four cruises (SO-226 [8], MsM39 [9], M113 [10] and M124 [11]) that were carried out between 2013 and 2016 (Fig 1). During the screening of the dried material, we targeted specimens that could be identified as belonging to one of the unsampled species. *Globorotaloides hexagonus* could be easily identified by its coarse wall texture and the presence of ~5 laterally compressed chambers in the final whorl. Specimens of this species were found frequently in subsurface samples collected in the Pacific Ocean during cruise SO226-3. The analyzed specimens covered a suite of different sizes. *Dentigloborotalia anfracta* is a morphologically distinct species easily distinguished by its kidney-shaped chambers. It also occurred frequently in the material from cruise SO226-3. The microperforate clade is subdivided into three genera [2]. Among these, the monotypic *Candeina nitida* is easy to distinguish even among juvenile specimens by irregular shape of chambers and presence of sutural apertures [12]. These characters allowed us to identify juvenile specimens of this species in warm-water samples from our collection with high level of fidelity. The remaining two genera are distinguished by the position of the primary aperture, being umbilical in *Globigerinita* and umbilical-extraumbilical in *Tenuitella*. This distinction could be followed in most cases. Among the three species of *Globigerinita*, we have for the first time extracted DNA from specimens that could be assigned to *G*. *minuta*. These specimens are distinguished from *G*. *glutinata* by round aperture with a lip (visible mostly in SEM images, see [2]), small and tightly coiled shell with a tendency towards a high-trochospiral coiling. The original description of the species mentions supplementary aperture, but these are obviously mistaken with accessory apertures of the bulla, a feature common to all *Globigerinita* species. The identification of *G*. *minuta* among the sequenced specimens proved to be most challenging of all the studied species. It was in the end only possible by SEM investigation of shells of multiple specimens revealing the distinct apertural shape. In contrast, *G*. *uvula* was consistently easy to identify by its highly trochospiral shell. Considering its good coverage by existing sequencing efforts [3], this species was not the primary target of our sampling. So far, no sequences of *Tenuitella* have been reported in the literature. Here we made use of on-board observations of *Tenuitella* in Pacific and South Atlantic cruises and identified *T*. *iota* as a form with four inflated chambers in the final whorl and *T*. *fleisheri* as a form possessing laterally compressed chambers. The third known species of *Tenuitella*, *T*. *parkerae* is easily distinguished by its radially elongated chambers. We have identified several specimens in our collections, but none yielded DNA. We did not identify specimens of any other of the unsampled species, such as *Berggrenia pumilio*. In addition to positively identified specimens, we also attempted to extract DNA from small specimens that appeared microperforate but could not be identified to a species level. The air-dried specimens were transferred into DNA extraction buffer in the laboratory. This material was supplemented with additional specimens collected during the MsM09 cruise [13] from the subarctic North Atlantic from 2008 where the specimens where identified and isolated into extraction buffer during the cruise. The collection details of all specimens are given in S1 Table.

### DNA extraction, amplification, sequencing and genotyping

**Fig 2:**
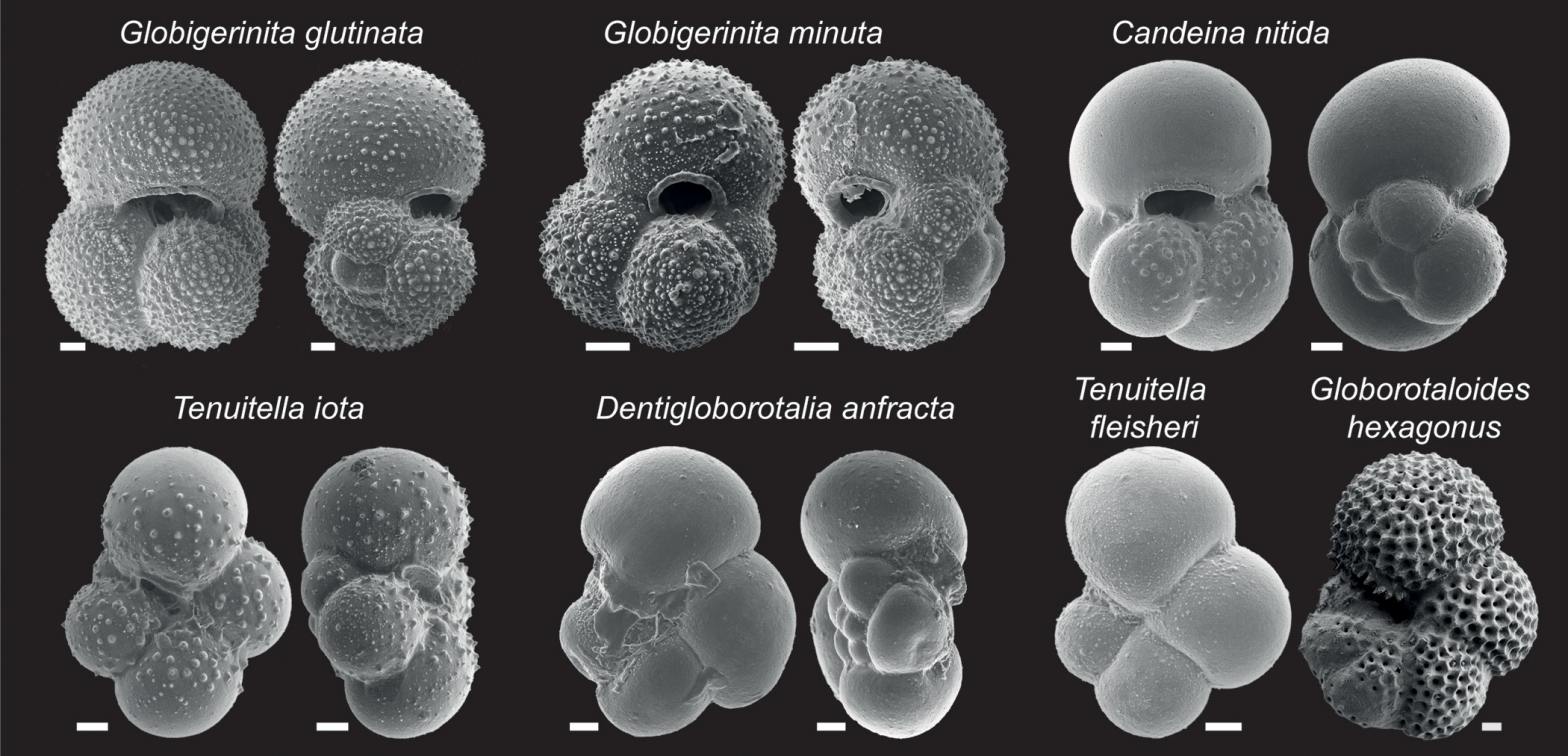
SEM pictures of the 7 morphological species that have been sequenced for this study. The scale bar represents 20 μm.

DNA extraction was performed using either the DOC protocol or the GITC* protocol (See Weiner et al. [7] for a detailed explanation of each protocol). The GITC* protocol bears the advantage of preserving the shell. A fragment of ~1000 bp located at the 5’-end of The 18S rDNA and located between stems 32 and 50 after Wuyts et al. [14] was amplified using the PHUSION polymerase (Fisher scientific) with the foraminifera specific primer pair S14F1 (5’-AAGGGCACCACAAGAACGC-3’) and 1528R (5’-TGATCCTTCTGCAGGTTCACCTAC-3’) [7]. The PCR amplification was carried out by mixing 1 μl of DNA extract with 0.4 μM of each primers, 3% of DMSO, 1X PHUSION buffer, 2.5 μM of MgCl_2_, 0.2 μM of dNTP, and 0.3 units of polymerase in a final volume of 15 μl. PCR amplification conditions were as follows: initial denaturation at 98°C for 30 seconds followed by 35 cycles at 98°C for 10 seconds, 65°C for 30 seconds and 72°C for 30 seconds and 2 minutes of final extension at 72°C. The obtained fragment covers the foraminifera specific metabarcode amplified in Morard et al. [4] and the hypervariable region V9 amplified by de Vargas et al. [6] (See S1 Fig). The resulting positive PCR products were purified using the QIAquick PCR purification kit (QIAGEN) and directly sequenced by an external provider (LGC Genomics, Berlin). To assess the extent of the intragenomic variability within the studied species, we cloned 31 specimens using the Zero Blunt TOPO PCR Cloning Kit (Invitrogen) with TOP10 chemically competent cells following manufacturer’s instructions. The specimens were selected to obtain at least two individuals of every morphospecies and potential cryptic species in our sampling (Table 1). Between 2 to 20 clones were sequenced per specimen using the same primers than for the original amplification. The chromatograms were carefully checked and only sequences of sufficient quality were retained and deposited on NCBI under the accession numbers MK499617 to MK500045. Short sequences (<200 bp) or sequences with ambiguous bases but still informative were used for genotyping (see below). These sequences are not reported in NCBI and have not been used for phylogenetic inference (Supplementary Material 1). SEM images of representative specimens for each morphological species analyzed (Fig 2) were obtained from material extracted using the GITC* protocol.

**Table 1:** Summary of the number of specimen analyzed, the number of sequences produced, the sequencing type and taxonomic units obtained for each morphospecies.

	*B*. *variabilis*	*D*. *anfracta*	*G*. *conglomerata*	*G*. *hexagonus*	*G*. *vivans*	*C*. *nitida*	*G*. *glutinata*	*G*. *minuta*	*G*. *uvula*	*T*. *fleisheri*	*T*. *iota*	Total
**Specimen sequenced**												
Specimen sequenced	45	6	3	12	2	34	134	33	144	12	16	**441**
N specimen directly sequenced	36	4	0	4	0	30	115	31	136	2	13	**371**
N specimen cloned	10	4	3	9	2	10	24	8	9	12	5	**96**
**N sequences**												
Database	76	0	9	24	5	18	114	3	167	29	8	**453**
New	0	36	0	40	0	76	116	81	4	37	57	**447**
Total	76	36	9	64	5	94	230	84	171	66	65	**900**
**Sequencing Type**												
Direct sequencing	36	4	0	4	0	30	115	31	136	2	13	**371**
Clone	40	32	9	60	5	64	115	53	35	64	52	**529**
**Curated Taxonomy**												
N unique BASETYPE	4	7	1	8	1	6	17	2	8	10	15	**79**
N MOTUs lvl-3	4	3	1	1	1	3	15	2	4	2	2	**38**
N MOTUs lvl-2	3	1	1	1	1	2	4	2	1	1	1	**18**
N MOTUs lvl-1	2	1	1	1	1	2	3	2	1	1	1	**16**

### Public databases

To obtain a maximum coverage of the distribution of each of the morphospecies, we retrieved all available sequences that could be assigned to the newly barcoded taxa from the PFR^2^ database v 1.0 [3]. This included 396 single-cell sequences assigned in PFR^2^ to *Globoquadrina conglomerata*, *Streptochilus globigerus*, *Galitelia vivans*, *Candeina nitida*, *Globigerinita glutinata*, *Globoigerinita uvula* and to 4 unindentified rotaliid species. We also retrieved 57 sequences from NCBI of the species *Bolivina variabilis* published in an earlier work [15]. We have observed inconsistencies in the labelling between the newly generated sequences and some sequences available in the public databases. We carried out a manual curation to correct the obvious misidentification and obtained a homogenized taxonomy at the morphospecies level (See Supplementary Material 1).

Foraminifera metabarcodes from plankton samples were retrieved from Morard et al. [4] as well as from the larger Tara Ocean project database published by de Vargas et al. [6] and available at http://taraoceans.sb-roscoff.fr/EukDiv/ from which all Operational Taxonomic Units (OTUs) assigned to foraminifera (Rank 3) were manually extracted. The geographic origin of the used samples is shown in Fig 1.

### Molecular taxonomy

To evaluate the extent of the genetic diversity among the newly barcoded taxa and to structure the observed variability into Molecular Operational Taxonomic Units (MOTUs) we applied the nomenclature system of Morard et al. [5]. The system is organized in three hierarchal levels below the morphospecies level based on the sequence fragment located at the 3’-end of the 18S rDNA between stems 32 and 50 [14] and is the barcode selected for benthic foraminifera [16]. The fragment harbors 6 variable regions, 3 being specific to foraminifera. To exclude potential sequencing errors when constructing the nomenclature, we retained only sequences for which the individual sequence pattern has been observed at least three times in our dataset. All distinct sequences in the resulting trimmed dataset are considered as *basetypes*.

The *basetypes* co-occurring within one or several individuals (because of intra-individual variability among tandem copies of the gene revealed by cloning) were assembled into *basegroups*, and constitute the lowest level of the nomenclature (MOTUs lvl-3). The variability observed within the basetype represents at least the intragenomic (intra-individual) variability and the variability observed among different basegroups is considered to represent at least the level of population variability (See Figs 5 and 6 of Morard et al. [5] for an illustrated example of the procedure). The second level of the nomenclature (MOTUs lvl-2) represents the level of biological species (or genotype) and the first level (MOTUs lvl-1) represents a major disruption in the genetic variability within a given morphospecies organized in monophyletic clusters (lineages) consisting of one or several genotypes. The levels 1 and 2 of the nomenclature were constructed using a combination of two automated delimitation methods, the Automated Barcode Gap Discovery method (ABGD) [17] and the Poisson Tree Process (PTP) [18]. Because both methods work better when the rates of evolution of the taxa analyzed are not too different, we separated the retained basetypes into two alignment sets based on the phylogenetic affinities of the selected morphospecies [4]. The basetypes of the morphospecies *Dendrigloborotalia anfracta*, *Globorotaloides hexagonus*, *Globoquadrina conglomerata*, *Bolivina variabilis*, *Galitelia vivans*, were grouped into the alignment”Basal” and the morphospecies *Candeina nitida*, *Globigerinita glutinata*, *Globigerinita minuta*, *Globigerinita uvula*, *Tenuitella fleisheri* and *Tenuitella iota* were grouped in the alignment “microperforates”.

**Fig 3:**
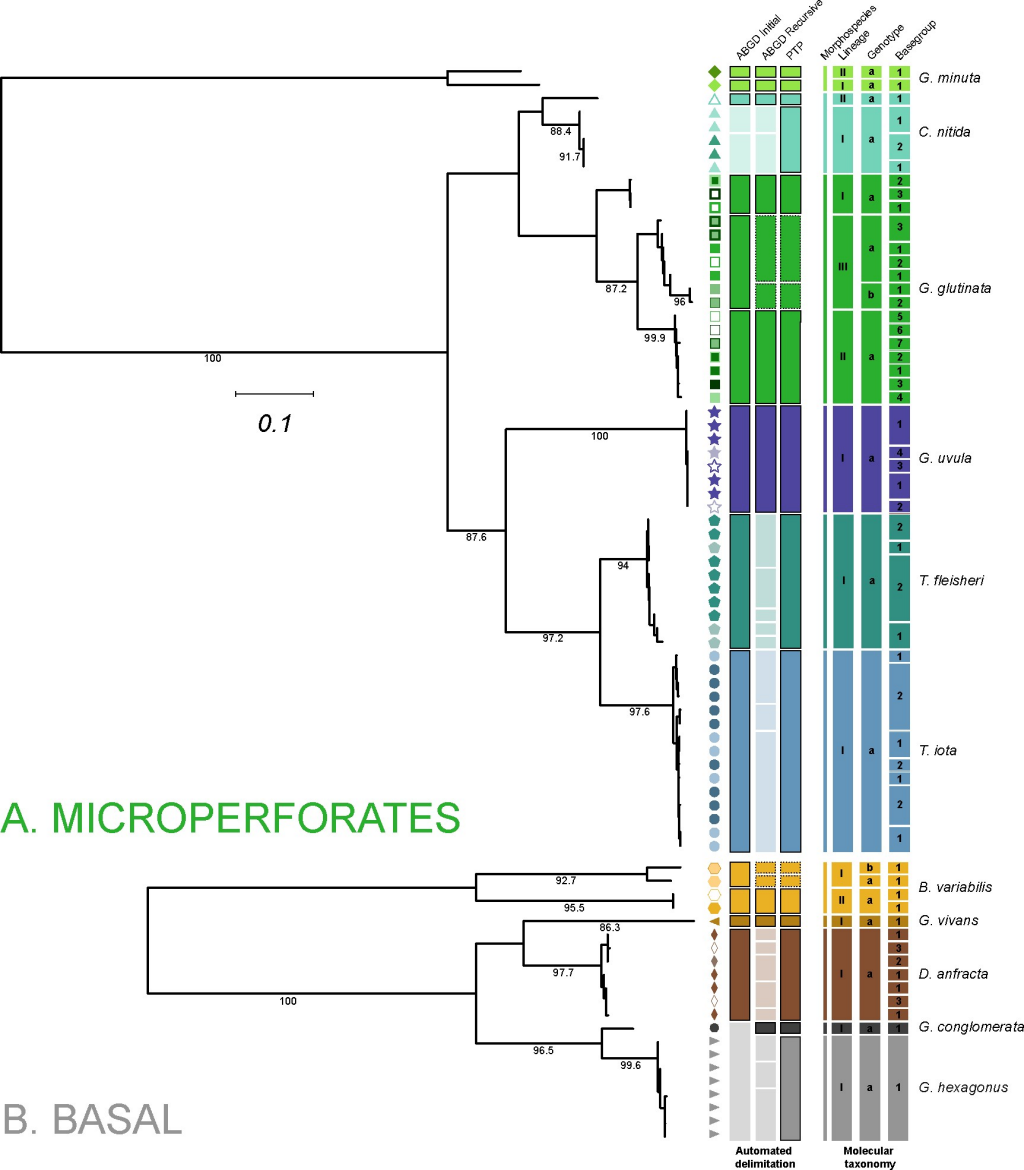
Molecular taxonomy of the (A) Microperforates and (B) Basal clades. Each branch represents a unique basetype, the symbol next to the branch represent the individual basegroup and the colors represent unique morphospecies. The first set of rectangles represent the three automated delimitation proposed by ABGD and PTP. In case that the proposed delimitations classify two sequences belonging to the same basegroup into two different partition, or if two sequences belonging to two different morphological species, the proposed delimitation is invalidated and shown in light color. When the delimitation are not invalidated, the coarsest partition is retained as MOTU level-1 and circled with a solid line, the finest partition is retained as MOTU level-2 and circled in dotted line. The resulting 3-rank molecular taxonomy is showed in the second set of rectangles.

Both files were automatically aligned with MAFFT [19] and a phylogenetic inference was calculated with 1000 non-parametric bootstrapping pseudo replicates based on a BioNJ starting tree using PhyML [20]. The best substitution models were selected using the Smart Model Selection [21] under Akaike Information Criterion and the model GTR+I+G was retained for the Basal tree and the GTR+I+G was retained for the Microperforate tree. The resulting trees are shown are shown on Fig 3.The same alignments were submitted to the online ABGD server (http://wwwabi.snv.jussieu.fr/public/abgd/abgdweb.html) using the Kimura K80 distance and default options. We retained the initial (coarsest delimitation) and recursive partition (finest delimitation) provided with the lowest prior intraspecific divergence. Prior to the PTP delimitation, we reiterated the two phylogenetic inferences but adding a sequence of the Nummulitidae species *Cycloclypeus carpentei* (accession number: AJ879133) as an outgroup because providing a rooted tree leads to better delimitation than unrooted tree with the PTP method. Choosing an outgroup for Microperforate and Basal groups is difficult because their exact phylogenetic position within the rotaliida is not known and the Basal group is polyphyletic, therefore we chose a benthic foraminifera that seemed to be reasonably distant from the planktonic foraminifera sequences and used it for the delimitation purpose only. We submitted the two resulting tree to the PTP server (http://species.h-its.org/), indicated *Cycloclypeus carpentei* as the outgroup and left the other parameters on default options.

We defined the MOTU lvl-2 as the finest delimitation proposed by ABGD and PTP and the MOTU lvl-1 as the coarsest. The proposed delimitations are retained as working hypothesis as long as two clones belonging to the same basegroup (and potentially to the same individual) are not attributed to different partition (oversplit) and if sequences belonging to different morphospecies are not grouped in the same partition (lumping). The delimitation proposed by ABGD and PTP as well as the retained delimitation are reported in Fig 3.

### Re-assignment of TARA reads and automated taxonomy

**Fig 4:**
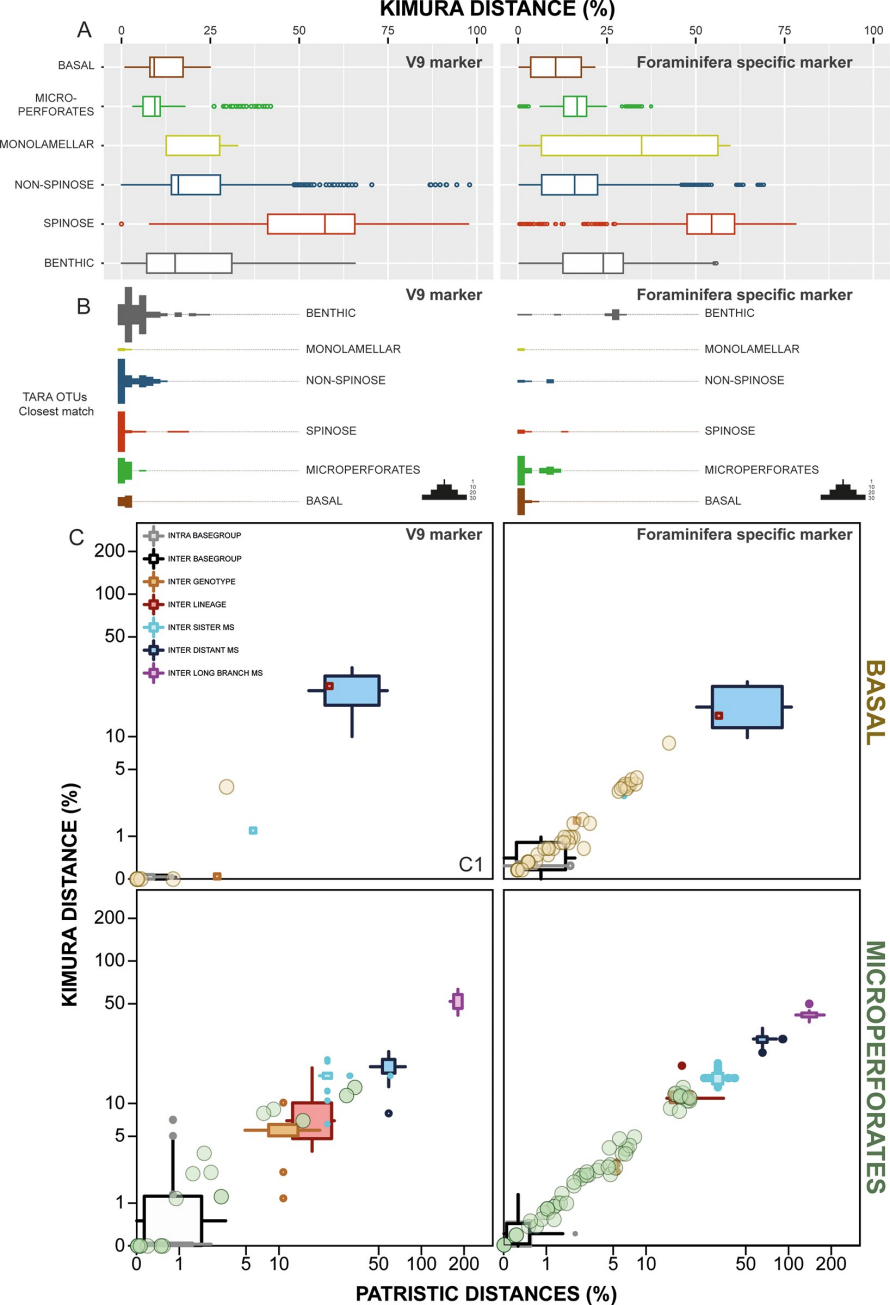
(A) Intra-clade variability observed on the V9 and foraminifera specific marker of the reference sequences based on Kimura genetic distance and (B) distribution of the closest match of OTUs of the 247 foraminiferal OTUs of the dataset of de Vargas et al. [6] and the 155 representative OTUs of Morard et al. [4]. The abscise axis are aligned with the (A) panel. (C) Integration of the Basal and Microperforates OTUs into the molecular taxonomy framework. The two-dimensional box plot represent the variability observed within each taxonomic level and the circled dots represent the lowest distance observed between the OTUs and the reference sequences.

Once the new barcode reference was assembled, including all the newly sequenced and reassigned sequences, we developed a two steps procedure to annotate the environmental OTUs from de Vargas et al. [6] and Morard et al. [4] and implemented them into the updated taxonomic framework. The marker amplified in de Vargas et al. [6] covers the single variable region V9 located at the end of the 18S and the marker amplified by Morard et al. [4] covers the variable regions 41F, 43E, 45E-47F as defined in Morard et al. [3]. First, we produced two separate alignments including the representative sequences of the 247 OTUs assigned to foraminifera by de Vargas et al. [6] and the 155 representative sequences for the foraminifera diversity as defined in Morard et al. [4]. These two alignments were extended by our updated barcoding dataset including 349 reference sequences for planktonic foraminifera and 231 benthic foraminifera sequences covering the main clades of benthic foraminifera and following the updated classification of the rotaliids [22,23]. The resulting “reference alignment” covered the regions amplified in de Vargas et al. [6] and Morard et al. [4] allowing a standardized comparison between the two separate markers. The two files were automatically aligned using MAFFT and we calculated the pairwise Kimura distances between the reference sequences of the two markers to identify distance gaps within and between clades of foraminifera (Fig 4A). Based on the closest pairwise distance between each of the OTUs and the reference, we could unambiguously identify the most likely attribution of OTUs to either one of the clades of planktonic foraminifera or to reveal an affinity to benthic foraminifera (Fig 4B).

**Fig 5:**
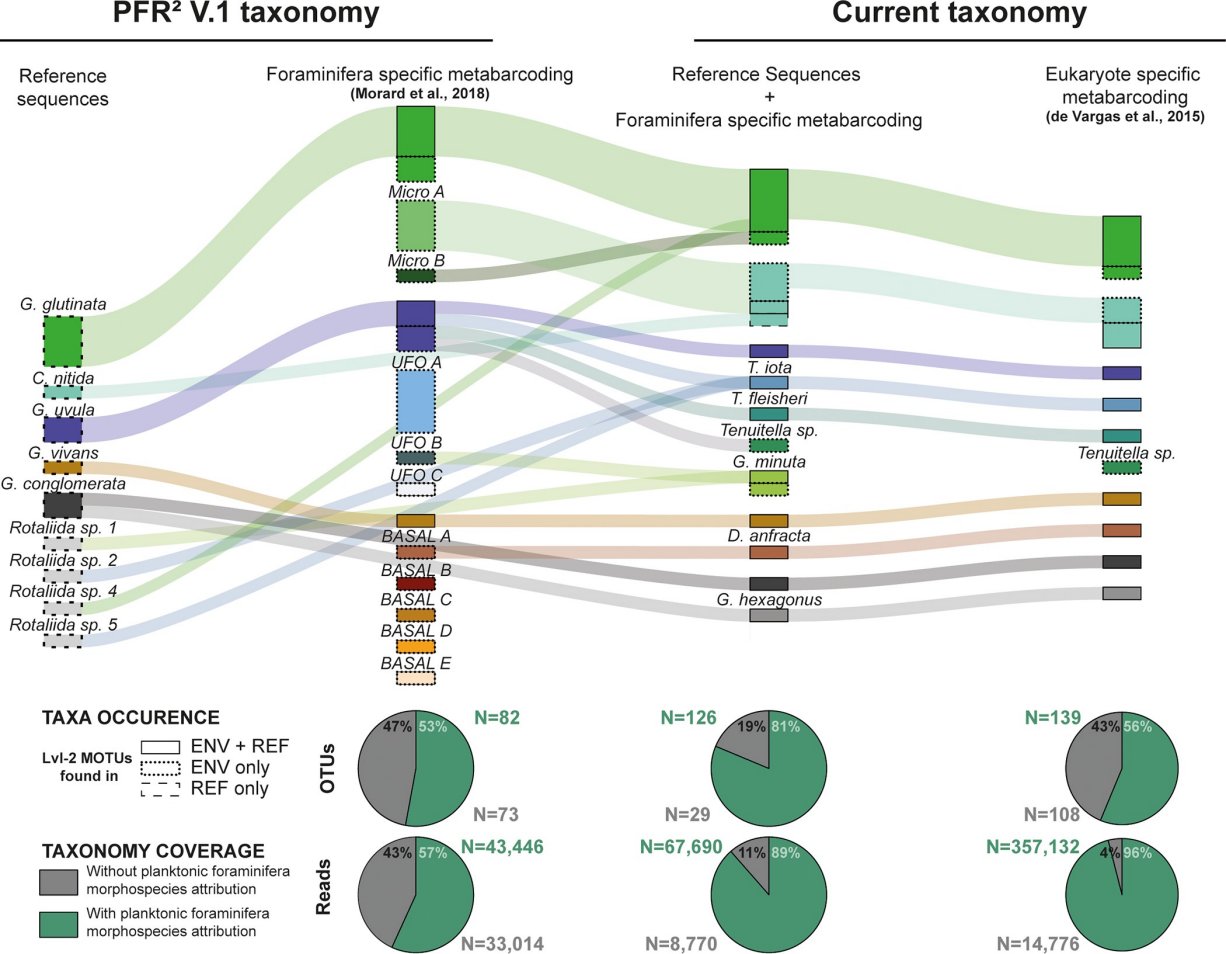
Taxonomic evolution and consistency across the datasets. The Sankey Diagram in the upper panel represents the evolution of the taxonomic units that have been either split or lumped with the taxonomic revision of the study. The size of the rectangles indicate the number of level-2 MOTUs identified in each morphological species, and the outline indicate in which dataset they have been identified (from either the reference sequences, environmental sequences or both). The lower panel indicate which portion of the diversity (Both for diversity–OTUs and volume—reads) could be attributed to morphological (barcoded) species of planktonic foraminifera.

**Fig 6:**
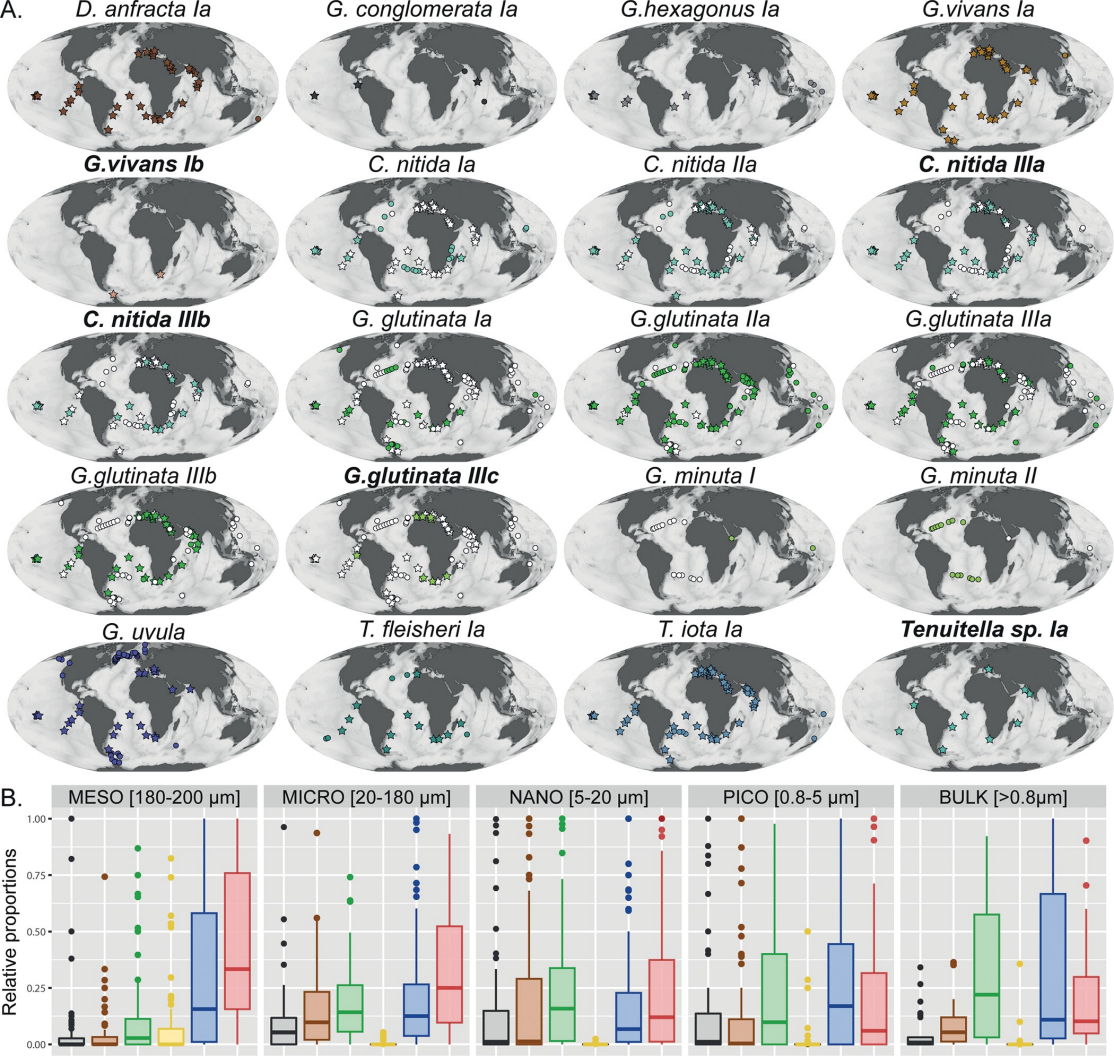
**(**A) Biogeographic distribution of the 20 MOTUs lvl-2 described in the study. The names in bold indicate where the taxa have been detected in environmental surveys only. The stars indicate the TARA ocean collection and the circle the single-cell collection. The symbol indicate where the morphospecies have been collected and are colored where the MOTU has been identified in the sample (Open symbols therefore indicate where the MOTUs have not been detected despite sampling). (B) Relative contribution of the 6 major clade of foraminifera in each of the size fraction represented in the TARA ocean collection.

In a second step, OTUs unambiguously attributed to the Basal or Microperforates clades (thus excluding the Macroperforate Globigerinidae, Globorotaliidae and Hastigerinidae and Benthic taxa) were aligned with sequences used to define the molecular taxonomy in our new barcoding reference (Fig 3). We aligned the sequences automatically with MAFFT and inferred a phylogeny using PhyML with aLRT branch support. We computed the pairwise patristic distances on the resulting trees together with the Kimura distances calculated on the metabarcode regions only. We compared the distance thresholds between the different taxonomic levels of the reference sequences to characterize the taxonomic structure of the environmental OTUs (Fig 4C). This allowed use to propagate the properties of our molecular nomenclature onto environmental sequences that were not identical to the reference, providing a means to explore the part of the diversity not covered by our single-cell survey within the same three-tier molecular taxonomy. As a result, we obtained a taxonomically homogenized dataset between environmental OTUs and single-cell barcodes that allowed us to ensure the taxonomic consistency across the dataset and estimate which portion of the diversity (OTUs) and volume (reads) of the metabarcoding dataset could be attributed to morphological species of planktonic foraminifera (Fig 5). Based on our integrated dataset, we unveiled the biogeographic pattern of the detected taxa (Fig 6A) and estimated the relative proportions of each of the foraminifera clade in the different plankton size fractions (Fig 6B).

## Results

### Extended barcode reference and molecular taxonomy

Out of the 544 specimens targeted for the extension of the barcode reference, 159 could be amplified successfully and yielded sequences long enough to allow taxonomic assignment. Among these, 27 sequences (mostly from small specimens that were not assigned to species level) could be identified as belonging to already known macroperforate taxa and were excluded from the dataset. The remaining 447 sequences derived from 132 specimens allowed us to expand the existing barcode reference by five morphospecies (Fig 2). The identification of these morphospecies is in all cases based on specimens that were positively identified as the given species prior to DNA extraction and for which the shells could have been examined afterwards by SEM (Fig 2). The availability of the new barcodes allowed us to carry out re-assignment of existing sequences in our databases and to reveal the identity of the DNA extracted, which could not be identified to species level. We observed that 29 sequences, which have been labelled in the PFR^2^ database as *Globigerinita uvula*, were nearly identical with new sequences extracted from specimens identified as *Tenuitella fleisheri*. Similarly, 24 sequences labelled in PFR^2^ as *Globoquadrina conglomerata* were identical to new sequences extracted from specimens identified as *Globorotaloides hexagonus*. We also noticed that the 2 sequences labelled in PFR^2^ as Rotaliida sp.1 were identical to new sequences from specimens whose taxonomic identity was revealed to be *Globigerinita minuta*, the 4 sequences labelled in PFR^2^ as Rotaliida sp.2 and the single sequence labelled as Rotaliida sp.5 were identical to new sequences extracted from specimens identified as *Tenuitella iota* and the 3 sequences labelled in PFR^2^ as Rotaliida sp. 4 could be shown to be identical to new sequences extracted from specimens identified as *Globigerinita glutinata*. All sequences named *Streptochilus globigerus* were renamed *Bolivina variabilis* after Kucera et al. [15]. The 61 DNA sequences extracted from “unidentified” small specimens could all be identified as belonging to sequences represented in the extended barcode dataset. Following the manual curation, all the sequences were merged into a dataset with internally consistent morphological taxonomy including 900 Sanger sequences derived from 441 individuals collected at 161 stations (Fig 1) and representing eleven morphotaxa (Table 1).

The molecular nomenclature was established separately for the microperforate clade and the taxa labelled as “basal” in Morard et al. [4]. Among the 190 sequences belonging to the Basal clade, 61 met the quality requirements to be used to define a total of 21 basetypes, which could be further organized into 10 basegroups. Likewise, from the 710 sequences belonging to the Microperforates clade, 233 qualified to define 58 basetypes that constituted 28 basegroups (Fig 3). Both phylogenetic inferences showed that in all cases basetypes assigned to the same morphospecies were monophyletic. We observed that *Globigerinita minuta* was highly divergent from other microperforate species. In stark contrast with other microperforate species, we did not observed any sign of intragenomic variability among the 33 specimen that were sequenced and from which 8 were cloned and 53 clones sequenced (Table 1). Prior to establishing the molecular nomenclature, we checked that the partition proposed by the three automated methods of delimitations did not violate any of the conditions (lumping of sequences belonging to different morphotaxa or splitting of basetypes belonging to the same basegroup). We invalidated the partitions proposed by “ABGD recursive” for *C*. *nitida*, *T*. *fleisheri*, *T*. *iota*, *D*. *anfracta* and *G*. *hexagonus* for oversplitting (Fig 3). Two of the partitions proposed by “ABGD initial” were rejected, one for splitting the *C*. *nitida* basegroup and one for lumping *G*. *hexagonus* and *G*. *conglomerata*. Based on the partitions that did not violate any of the conditions, our approach did not identify any MOTUS at levels 1 and 2 in *G*. *hexagonus*, *G*. *conglomerata*, *G*. *vivans*, *D*. *anfracta*, *T*. *iota*, *T*. *fleisheri* and *G*. *uvula*. We identified two lineages (MOTUs lvl-1) in *G*. *minuta* and *C*. *nitida* each comprising a single genotype. We identified 2 and 3 lineages in *B*. *variabilis* and *G*. *glutinata* respectively, and only a single linage in each species had two genotypes.

### Taxonomic assignment of metabarcode OTUs

The re-analysis of the 155 representative OTUs of Morard et al. [4] attributed 62 OTUs to the Microperforates clade, 41 to the Basal clade, 23 to the Non-Spinose clade, 12 to the Benthic clade, 15 to the Spinose clade and 2 to the Monolamelar clade. The same approach applied on the 247 foraminifera OTUs identified in de Vargas et al. [6] showed that 107 were attributed to Benthic foraminifera clade, 60 to Non-Spinose clade, 34 to Spinose clade, 30 to Microperforates clade, 14 to Basal clade and 3 to the Monolamellar Clade (Fig 4A and 4B). Among the OTUs attributed to the Basal and Microperforates clades in both datasets, none had a genetic distance greater that the level characterized by congeneric morphospecies (Fig 4C).

The re-assessment of the diversity of representative OTUs of Morard et al. [4] showed that 28 of the 41 OTUs of the Basal clade and 37 of the 62 OTUs of the Microperforate clade had a distance compatible with intra or inter basegroup variability observed in the reference sequences, and thus are likely to not represent additional diversity. We identified three clusters of sequences in the 13 remaining OTUs of the Basal clade that were compatible with the level of congeneric species to *D*. *anfracta* and *G*. *vivans*. Among the 35 OTUs allocated to the microperforates with a distance greater than the inter-basegroup distances, 13 were allocated to *C*. *nitida*, 7 to *Tenuitella* and 5 to *G*. *glutinata*, and represented potentially 3 additional genotypes for *C*. *nitida*, 2 for *G*. *glutinata* and a *Tenuitellid* morphological species not covered by our barcoding survey. To avoid potential confusion, we chose not give names to sequence clusters only detected in the environmental samples from Morard et al. [4] and reserve the naming to sequences in the dataset of de Vargas et al. [6] with a larger geographical coverage (see below).

The same procedure applied on the foraminifera OTUs in the dataset of de Vargas et al. [6] showed that among the 8 and 34 OTUs belonging to the Basal and Microperforates clade, 7 and 26 had a genetic distance with the reference sequences compatible with intra- or inter-basegroup variability, and thus likely to not reflect additional biological diversity. Among the remaining OTUs, one Basal and one Microperforate OTU had a distance compatible with an additional genotype, sister to *G*. *vivans* Ia (thus names *G*. *vivans* Ib) and *G*. *glutinata* IIIa/IIIb (thus named *G*. *glutinata* IIIc). Four Microperforates OTUs had a distance compatible with an additional lineage of the morphospecies *C*. *nitida*, and were structured in two clusters likely to represent two distinct genotypes (thus named *C*. *nitida* IIIa and IIIb). Two OTUs had a distance sufficient to represent a sister species to *Tenuitella fleisheri*, and were therefore named *Tenuitella sp*. Ia. Finally, the last OTU with a large distance seemed to be a chimera after closer examination and was not considered for the rest of the analysis.

### Consistency of diversity revealed by metabarcoding datasets

The addition of small taxa in our reference database associated with the revision of the taxonomy permitted us to redraw the boundaries and re-allocate the unknown diversity identified in Morard et al. [4] while constraining the foraminiferal diversity found in the Tara Ocean metabarcode of de Vargas et al. [6] (Fig 5). This had the additional advantage of an expansion of the biogeographic analysis to 47 globally distributed stations compared to the 8 stations from the South Atlantic analyzed by Morard et al. [4]. The addition of new barcodes showed that the environmental OTUs initially clustered as “Micro-A”, “Micro-B” and “UFO-B” in Morard et al. [4] actually belonged to *C*. *nitida*, *G*. *glutinata* and *G*. *minuta* respectively. The largest cluster of OTUs named “Basal-A” corresponded to the newly barcoded *D*. *anfracta* and finally, the clade labelled as *G*. *uvula* comprising four lineages was revealed as an aggregation of *G*. *uvula* and three morphological species of *Tenuitella*. Despite our barcoding efforts, we did not find a fit in our reference dataset for the environmental clusters UFO-A, UFO-C, Basal-B, Basal-C, Basal-D and Basal-E. Nonetheless, our additional barcoding effort targeted towards small size fraction characterized a much larger portion of the environmental dataset with over 81% of the OTUs diversity and 89% of read volume allocated to morphological species against 53% and 57% prior to the current study. The extension of our barcoding reference dataset to the larger dataset of de Vargas et al. [6] identified the same level of diversity in the Microperforates and Basal clades. However, only 56% of the OTU diversity in this dataset could be attributed to planktonic foraminifera morphological species despite the fact that 96% of the read volume of the dataset is characterized. This is due to the occurrence of a diverse consortium of OTUs in this dataset that represent a modest portion of the reads and has an affinity to various groups of benthic foraminifera (Fig 4B).

### Biogeography and abundance of the small and neglected taxa

Among the eleven morphospecies belonging to the “small and neglected” Microperforates and Basal groups, only four showed clear evidence for cryptic diversity (*G*. *vivans*, *C*. *nitida*, *G*. *glutinata* and *G*. *minuta*). In the case of *G*. *glutinata* and *C*. *nitida* we found no clear evidence of biogeographic or ecological differentiation among their constitutive genotypes even if *G*. *glutinta IIa* seemed to be the most widespread (Fig 6). The two lineages of *G*. *minuta* appear to be endemic with the lineage I being found in the Indo-Pacific and the Lineage II in the Atlantic, but the low number of samples in the Indo-Pacific prevents us from drawing a definitive conclusion. Finally, the potential second genotype of *G*. *vivans* occurred only at two Tara ocean station in the Southern Ocean. Among the seven taxa without genetic diversity at MOTU level 1 and 2, five appear ubiquitous, and *G*. *conglomerata* and *G*. *hexagonus* appear to be confined to low latitudes.

Compared to the 88,734 reads from the eight South Atlantic stations analysed by Morard et al. [4], the global Tara Ocean dataset of de Vargas et al. [6] yielded 371,908 foraminifera reads (Fig 5), distributed globally (Fig 1), providing a substantially larger baseline to assess differences in the abundance of the different groups of foraminifera encountered in the plankton. First, we observe that the high diversity of OTUs that could be assigned to benthic foraminifera constituted only a small portion of the reads extracted from the plankton samples (4% on the total dataset, Fig 5) which was the lowest on average in each samples (Fig 6B). Among the reads that could be assigned to one of the planktonic groups, the “small and neglected” Basal and Microperforates clades were most abundant, dominating the micro, nano, pico, and bulk size fractions. The Monolamellar taxa had the lowest contribution in all except the meso size fractions and the Spinose and Non-spinose clades were dominating the meso size fraction.

## Discussion

In this study we followed the hypothesis that the unassigned lineages belong to the group of small and obscure taxa that have been formally described but are recorded so rarely that their ecology, abundance, and genetic identity remain poorly constrained. Our barcoding effort targeted on small specimens and on the microperforate clade and revealed that this hypothesis is likely correct. A large portion of the uncharacterized diversity identified by Morard et al. [4] could be allocated to new barcodes representing small and obscure taxa. Although we still cannot characterize all the environmental OTUs, we show that by adding only five additional morphological species to our barcode catalog and extending the coverage in the microperforate clade allowed us to reduce the unassigned diversity in the environmental dataset from Morard et al. [4] from almost half to less than one fifth and the number of reads associated with the unassigned diversity from 43% to mere 11% (Fig 6). This result supports our hypothesis that the two centuries of taxonomic work on modern planktonic foraminifera resulted in a framework which captures the entire morphological diversity of the group, but the associated ecological research underestimated the role of “obscure” taxa.

These results document how incompleteness and incorrect entries of a barcoding reference may propagate into the interpretation of diversity patterns. Next to revealing some of the “unknown” lineages as belonging to the newly barcoded taxa, our analysis shows that several of the OTUs were initially “unassigned” because of incomplete barcode coverage within genetically diverse lineages. This is reflected in the initial mislabeling of lineages “Micro-A” and “Micro-B” that are now assigned to *G*. *glutinata* and *C*. *nitida*. Conversely, the diversity within the *Globigerinita uvula*/*Tenuitela* clade was initially interpreted too conservatively and in the absence of the appropriate barcode, sequences of *Tenuitella* were labelled as genotypes of its sister clade *Globigerinita uvula* (Fig 6). These examples illustrate that the correct interpretation of metabarcoding datasets requires robustly curated barcoding databases with appropriate coverage [24,25], and that barcoding efforts should continue in pace with the growing body of environmental meta–omics datasets.

The new planktonic foraminifera barcoding reference covers now 37 of the 47 formally described species. The 10 species that remain to be barcoded represent only ~20% of the morphological diversity, which is comparable to the 19% of the OTU diversity identified in Morard et al. [4] that is still unassigned after our re-assessment. We therefore speculate that there are no taxonomically unknown planktonic foraminifera lineages occurring in metabarcoding dataset. This hypothesis is supported by applying the new reference barcode on the much more extensive metabarcode of foraminifera sequences extracted from the TARA Oceans dataset of de Vargas et al. [6], where 96% of the reads could be attributed to known lineages of planktonic foraminifera (Fig 6). The remaining 4% of the reads show affinities to various groups of benthic foraminifera and are partitioned into a larger number of OTUs. These reads likely represent a mixture of planktonic foraminifera morphospecies without barcodes and specimens of benthic foraminifera present in the plankton due to a combination of passive advection in coastal regions or as reproductive strategy [15].

Attributing a large part of the previously unassigned lineages to obscure taxa like *D*. *anfracta* and the microperforate *G*. *minuta* or *Tenuitella* implies that the ecological importance of these taxa has been underestimated. Their high numerical abundance in the South Atlantic metabarcode analysed by Morard et al. [4] is consistent with their high abundance in the global TARA Oceans dataset (Fig 6). Although the two datasets are based on a different marker region and are thus subject to potentially different PCR bias, the comparison clearly reveals that the “obscure” taxa are prominent in metabarcodes from size fractions below the threshold of common micropaleontological studies (< 0.180 mm; Fig 6) and that the microperforates clade seems to be the most abundant on average across all size fraction (Fig 6). This is in agreement with observation made by Brummer et al. [12] who analyzed planktonic foraminifera with classical taxonomy across an broad size range and concluded that, unexpectedly, “small” species may account for as much as a third of the community composition. This number is remarkably similar to the relative proportions of the microperforate and basal clades among the reads in the bulk sample, demonstrating that morphology and molecules results meet if the appropriate reference database and taxonomy are applied on–omics data.

An analysis of the occurrence pattern of the newly identified and characterized “obscure” and microperforate taxa reveals further unexpected patterns. First, among the newly barcoded eleven morphological species within the group, seven lacked cryptic genetic diversity (Fig 3) and this pattern remains even when the TARA Oceans metabarcode is analyzed, revealing only one potential additional cryptic species in *G*. *vivans* and a new *Tenuitella* lineage, which likely represent *T*. *parkerae*. The 18S has been shown to be variable enough among all clades of planktonic foraminifera [5,26,27], including in the basal clade with *Bolivina variabilis* [15], and in the microperforate clades with *Globigerinita glutinata*, *Globigerinita minuta* and *Candeina nitida* to reveal cryptic diversity. Therefore, the apparent absence of cryptic diversification for seven species is astonishing because so far only the spinose species *Trilobatus sacculifer* was shown to lack cryptic diversity [28,29]. Second, we observe that the majority of the “small and obscure” taxa are cosmopolitan (Fig 6). Finally, in taxa with cryptic genetic diversity like *Globigerinita glutinata* and *Candeina nitida*, the constituent genotypes show no clear biogeographic or ecological differentiation (Fig 6A). It seems that the previous focus on the macroperforate clades may have also distorted our view on the diversity of ecological strategies in planktonic foraminifera. The broad biogeographic range and limited diversity of the newly assigned “small” lineages may indicate that their speciation is not driven by the conquest of new habitats or biogeographic ranges.

## Conclusion

Our study confirms that the diversity of planktonic foraminifera is limited and likely completely captured by morphological taxonomy. The barcoding of only five small and obscure species permitted to re-assign nearly half of the unknown diversity of the first metabarcoding survey of planktonic foraminifera and we conjecture that only a modest effort will be necessary to complete the reference database to a degree allowing unambiguous attribution of all metabarcodes. The cosmopolitan distribution of the “small and obscure” taxa, their modest cryptic diversification, and their abundance revealed by the metabarcoding dataset speak for opportunistic behavior and a much larger ecological significance of this group than previously assumed.

## Supporting information

S1 Table Metadata and taxonomy of the Sanger sequences used in the study. (XLSX)

S1 Fig Scheme of the ribosomal DNA with its variable (gray) and conserved (white) regions. The fragment mostly analyzed in planktonic foraminifera, located at the 3′ end of the SSU up to the ITS regions, is shown in more detail (variable regions as lines and conserved regions as boxes) and includes the position of the fragment amplified in the present study and in Morard et al. [4] and de Vargas et al. [6]. The figure is modified from Weiner et al. [7].
(TIF)
