# Supplementary figures and images for: Unassigned diversity of planktonic foraminifera from environmental sequencing revealed as known but neglected species
Source: PLoS One. 2019 Mar 21;14(3):e0213936. doi: 10.1371/journal.pone.0213936 (PMC6428320; doi:10.1371/journal.pone.0213936)

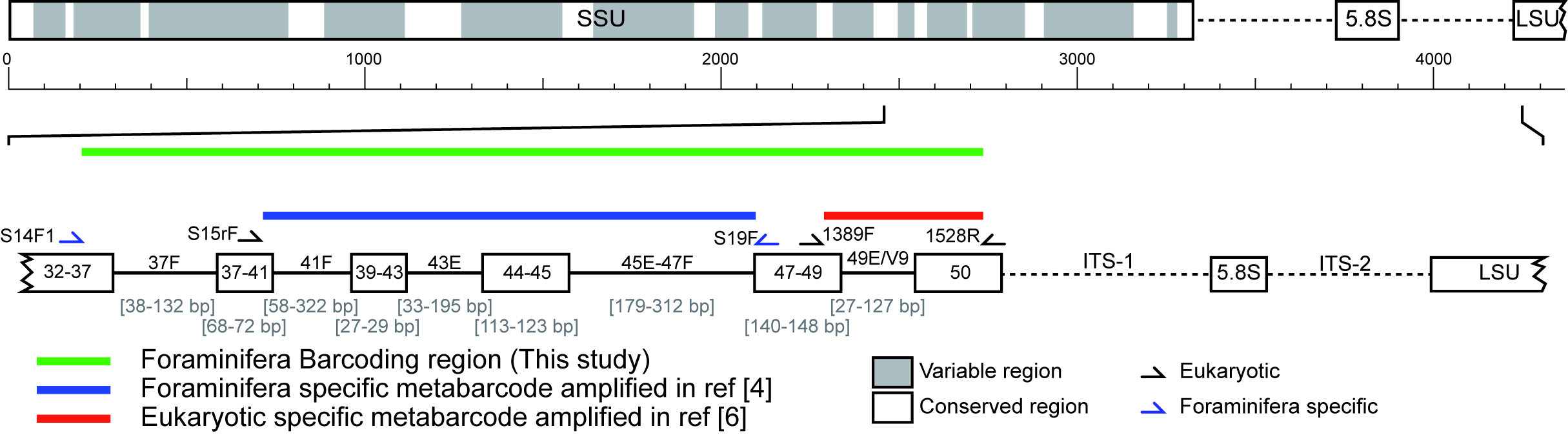

Supplement: S1 Fig — The fragment mostly analyzed in planktonic foraminifera, located at the 3′ end of the SSU up to the ITS regions, is shown in more detail (variable regions as lines and conserved regions as boxes) and includes the position of the fragment amplified in the present study and in Morard et al. [4] and de Vargas et al. [6]. The figure is modified from Weiner et al. [7]. (TIF) [file pone.0213936.s002.tif]
